# Association of parental favouritism in childhood and depression in old age: a longitudinal survey

**DOI:** 10.7189/jogh.15.04343

**Published:** 2025-12-22

**Authors:** Dongxu Li, Zhengrong Li, Weile Zhang, Hongying Ge, Min Su, Xi Guo

**Affiliations:** 1 School of Public Health, Jining Medical University, Jining, China; 2 School of Public Management, Inner Mongolia University, Hohhot, China; 3 School of Economics and Management, Inner Mongolia University of Technology, Hohhot, China

## Abstract

**Background:**

The global prevalence of depression is on the rise, and has evolved into a major public health concern. Given that parental favouritism in childhood exerts a long-term impact on children’s mental health, we aimed to explore its association depression in old age.

**Methods:**

We retrieved 19 180 data points from 3836 individuals aged 60 years and over from the China Health and Retirement Longitudinal Study 2011, 2013, 2015, 2018, and 2020 waves, among whom depression was measured using the Centre for Epidemiological Studies Depression Scale. We used logistic analysis to determine the relationship between parental favouritism in childhood and depression in old age, and the bootstrap method to analyse the mediating role of smoking, drinking, socialising, exercising, and sleep duration.

**Results:**

Approximately 17% of older adults experienced parental favouritism during childhood. We found that parental favouritism significantly increased the probability of depression by 29.9% (*β* = 0.299; 95% confidence interval (CI) = 0.145, 0.453), as did mother’s favouritism by 28.4% (*β* = 0.284; 95% CI = 0.117, 0.450), and father’s favouritism by 23.6% (*β* = 0.236; 95% CI = 0.058, 0.415). Socialising (*β* = −0.0018; 95% CI = −0.0028, −0.0009), exercising (*β* = 0.0009; 95% CI = 0.0003, 0.0017), and sleep duration (*β* = 0.0046; 95% CI = 0.0015, 0.0076) mediated this relationship. An analysis of heterogeneity by gender found that women are more influenced by parental favouritism.

**Conclusions:**

Parental favouritism in childhood significantly predicts the probability of depression in old age. Promoting socialising, exercising, and sleep duration could help to alleviate this issue. We also noted that women are more influenced by parental favouritism. These findings provide guidance for targeted interventions, such as mental health screening and promotion of healthy lifestyles.

The prevalence of depression is on the rise globally [1]. Studies have shown that familial experiences have a particular impact on the development of depression [2], with parental behaviours, including parental favouritism, having a clear effect on children’s psychological health [3,4]. Parental favouritism refers to parents’ being more active in bringing up one or more of their children than another or others [5]. This has been shown to negatively impact children’s development [6,7], including in terms of well-being [8], sibling relationships [9], parent-child relationships [10], and self-esteem [11]. Importantly, it may also increase the probability of developing depression [12]. In this sense, previous research has found that the effects of parental favouritism on children’s mental health persist into adulthood [13,14].

However, most of these studies used cross-sectional data and small sample sizes, while the mechanism of action of parental favouritism on depression in old age remains to be explored. Therefore, using data from five surveys conducted over a 10-year period, we investigated the relationship between parental favouritism and depression in old age and the underlying mechanisms, with the aim of providing empirical evidence for the promotion of mental health in this population. Specifically, we sought to explore the association between experiencing parental favouritism in childhood and depression in old age, the mediators of this relationship, and how it differs between gender heterogeneity.

## METHODS

### Data sources

The China Health and Retirement Longitudinal Study (CHARLS) data was a nationally representative cohort study of middle-aged and older adults (45 years or older) in China, and was designed to assesses issues related to population ageing and health among respondents. It used a probability proportional to size sampling technique, with a baseline survey in 2011 and follow-ups in 2013, 2015, 2018, and 2020. The Life Course Survey of Chinese Residents was concurrently conducted in 2014 as a special sub-survey of the CHARLS. Adopting a retrospective approach, it documented the life experiences of CHARLS respondents since birth in order to explore factors influencing health and life outcomes in old age.

**Figure 1:**
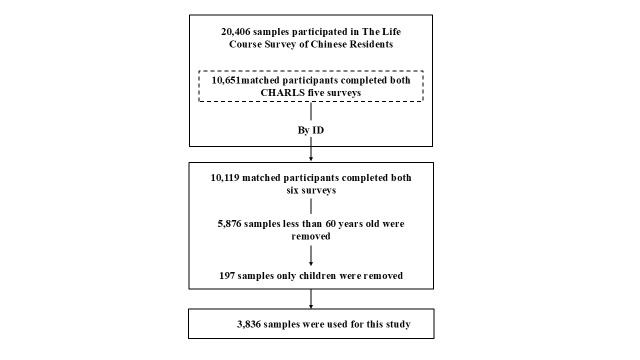
Sample selection flowchart.

Based on the sample ID, we horizontally matched data from the 2011, 2013, 2015, 2018, and 2020 waves to identify 10 651 individuals who participated in all five surveys. The Life Course Survey of Chinese Residents (n = 20406) was conducted based on the CHARLS platform, and we identified the same cohort of respondents through ID matching, yielding data on childhood parental favouritism for 10 119 individuals. After filtering out individuals aged <60 years (n = 5876) and only children (n = 197), we retained 3836 individuals for analysis (**Figure 1**).

### Variable selection and assignment

The dependent variable in this study was depression in older adults, assessed in the CHARLS using the Centre for Epidemiological Studies Depression Scale (CES-D10), which consists of 10 questions about the respondent’s experience of feeling annoyed, having difficulty concentrating, and other conditions during the past week. The resulting scores range from 0 to 30. Here, we considered a CES-D10 score of <10 as no depression and assigned it a value of 0, and a score ≥10 as indicative of depression, assigning it a value of 1 [15,16].

The independent variable in this study was whether the respondents experienced parental favouritism during childhood. Through the Life Course Survey of Chinese Residents, participants were asked ‘Did your female guardian treat your siblings better than you when you were growing up?’ and ‘Did your male guardian treat your siblings better than you when you were growing up?’. We assigned a value of 1 if there was an experience of parental favouritism, and a value of 0 otherwise.

Based on previous research [17-19], we included the following control variables: smoking (no = 0, yes = 1), drinking (no = 0, yes = 1), exercising (no = 0, yes = 1), socialising (no = 0, yes = 1), sleep duration (continuous variable), gender (women = 0, men = 1), age (continuous variable), income (continuous variable), area of residence (rural = 0, urban = 1), marital status (unmarried = 0, married = 1), educational level completed (primary school or below = 0, middle school or above = 1), chronic disease (no = 0, yes = 1), and health insurance (no = 0, yes = 1). We also controlled for time and region fixed effects.

### Statistical methods

Since the dependent variable was categorical, we used logistic regression analysis to investigate the relationship of parental favourism and depression, and utilised bootstrapping to analyse the mediating role of smoking, drinking, socialising, exercising, and sleep duration therein. For descriptive statistics, we expressed categorical variables as counts and percentages, and continuous variables as means and standard deviations (SDs). We performed the analyses in Stata, version 15.1 (Stata Corp, College Station, Texas, USA). The significance level for all hypothesis tests was set at 0.05.

## RESULTS

### Descriptive statistics

A total of 640 (16.68%) individuals in our sample experienced parental favouritism, while 3196 (83.316%) did not. There were 1989 (51.85%) women and 1847 (48.15%) men, with an average age of 67 years in 2011 and an average income of CNY 9287. A total of 2608 (67.99%) and 1228 (32.01%) individuals resided in rural and urban areas, respectively; 3187 (83.081%) were married; while 3159 (82.35%) and 677 (17.65%) had a primary school education or middle school education and above, respectively. Lastly, there were 2813 (73.33%) individuals with chronic diseases and 3669 (95.65%) individuals covered by health insurance (**Table 1**).

**Table 1:** Descriptive statistical analysis at baseline (2011)*

	Total (n = 3836)	Parental favouritism (n = 640, 16.68%)	No parental favouritism (n = 3196, 83.32%)	*P*-value
**Gender**				
Female	1989 (51.85)	335 (52.34)	1654 (51.75)	
Male	1847 (48.15)	305 (47.66)	1542 (48.25)	
**Age in years, x̄ (SD)**	66.585 (5.83)	66.077 (5.70)	66.687 (5.85)	
**Income, x̄ (SD)**	9286.546 (32 152.26)	11 954.633 (38 997.26)	8752.260 (30 577.38)	
**Area of residence**				<0.001
Rural	2608 (67.98)	378 (59.06)	2230 (69.77)	
Urban	1228 (32.01)	262 (40.94)	966 (30.23)	
**Marital status**				0.586
Married	3187 (83.08)	527 (82.34)	2660 (83.23)	
Unmarried	649 (16.92)	113 (17.66)	536 (16.77)	
**Educational level**				<0.001
Primary school or below	3159 (82.35)	489 (76.41)	2670 (83.54)	
Middle school or above	677 (17.65)	151 (23.59)	526 (16.46)	
**Chronic disease**				0.452
Yes	2813 (73.33)	477 (74.53)	2336 (73.09)	
No	1023 (26.67)	163 (25.47)	860 (26.91)	
**Health insurance**				0.977
Yes	3669 (95.65)	612 (95.63)	3057 (95.65)	
No	167 (4.35)	28 (4.37)	139 (4.35)	

SD – standard deviation, x̄ – mean

*Values are presented as n (%) unless specified otherwise.

### Impact of parental favouritism on depression

The regression results showed that parental favouritism significantly increased the probability of depression by 29.9% (*β* = 0.299; 95% confidence interval (CI) = 0.145, 0.453), as did mother’s favouritism by 28.4% (*β* = 0.284; 95% CI = 0.117, 0.450) and father’s favouritism by 23.6% (*β* = 0.236; 95% CI = 0.058, 0.415). We performed regression analyses by replacing the depression dummy variable with depression scale scores; the results showed that parental favouritism was significantly associated with increased depression scores (*β* = 0. 662; 95% CI = 0.327, 0.997) (**Table 2**).

**Table 2:** Analysis of the effect of parental favouritism on depression

	*β* (95%CI)	*P*-value	*β* (95%CI)	*P*-value	*β* (95%CI)	*P*-value	*β* (95%CI)	*P*-value
**Parental favouritism**	0.299 (0.145, 0.453)	<0.001					0.662 (0.327, 0.997)	<0.001
**Mother’s favouritism**			0.284 (0.117, 0.450)	0.001				
**Father’s favouritism**					0.236 (0.058,0.415)	0.010		<0.001
**Gender (female as reference)**	−0.548 (−0.669, −0.426)	<0.001	−0.547 (−0.668, −0.425)	<0.001	−0.553 (−0.674, −0.431)	<0.001	−1.483 (−1.742, −1.223)	<0.001
**Age**	−0.036 (−0.046, −0.025)	<0.001	−0.036 (−0.047, −0.026)	<0.001	−0.036 (−0.050, −0.030)	<0.001	−0.017 (−0.038,0.005)	0.140
**Income**	−0.029 (−0.043, −0.015)	<0.001	−0.029 (−0.043, −0.015)	<0.001	−0.029 (−0.043, −0.014)	<0.001	−0.059 (−0.083, −0.034)	<0.001
**Area of residence (rural as reference)**	−0.489 (−0.619, −0.359)	<0.001	−0.484 (−0.614, −0.354)	<0.001	−0.481 (−0.610, −0.351)	<0.001	−1.261 (−1.535, −0.987)	<0.001
**Marital status (unmarried as reference)**	−0.195 (−0.321, -0.068)	0.002	−0.195 (−0.321, −0.069)	0.002	−0.199 (−0.325, −0.072)	0.002	−0.636 (−0.885, −0.387)	<0.001
**Educational level completed (primary school or below as reference)**	−0.347 (−0.513, −0.181)	<0.001	−0.348 (−0.515, −0.183)	<0.001	−0.340 (−0.506, −0.174)	<0.001	−1.301 (−1.645, −0.957)	<0.001
**Chronic disease (no as reference)**	0.722 (0.586, 0.857)	<0.001	0.724 (0.589, 0.860)	<0.001	0.721 (0.586, 0.857)	<0.001	1.555 (1.274, 1.836)	<0.001
**Health insurance (no as reference)**	−0.053 (−0.229, 0.123)	0.554	−0.053 (−0.229, 0.122)	0.551	−0.052 (−0.228, 0.124)	0.563	−0.254 (−0.570, 0.061)	0.114
**Time effect**	Yes		Yes		Yes		Yes	
**Regional effect**	Yes		Yes		Yes		Yes	
**Wald’s χ^2^**	629.14		626.15		621.95		1001.71	
***P*-value**	<0.001		<0.001		<0.001		<0.001	
**n**	19 180		19 180		19 180		19 180	

CI – confidence interval, *β* – correlation coefficient

#### Mediating effect of parental favouritism on depression

Socialising (*β* = −0.0018; 95% CI = −0.0028, −0.0009), exercising (*β* = 0.0009; 95% CI = 0.0003, 0.0017), and sleep duration (*β* = 0.0046; 95% CI = 0.0015, 0.0076) could act as mediating variables, meaning that parental favouritism increased the likelihood of depression by decreasing the probability of the individual experience these three factors (**Table 3**).

**Table 3:** Mediation effect test: bootstrap methods

	*β* (95% CI; bias-corrected 95% CI)	*P*-value
**Parental favouritism – smoking – depression**	0.0001 (−0.0001, 0.0003; −0.0000, 0.0004)	0.756
**Parental favouritism – drinking – depression**	−0.0001 (−0.0006, 0.0002; −0.0007, 0.0001)	0.338
**Parental favouritism – socialising – depression**	−0.0018 (−0.0029, −0.0010; −0.0028, −0.0009)	<0.001
**Parental favouritism – exercising – depression**	0.0009 (0.0002, 0.0016; 0.0003, 0.0017)	0.014
**Parental favouritism – sleep duration – depression**	0.0046 (0.0015, 0.0076; 0.0015, 0.0076)	0.003

CI – confidence interval, *β* – correlation coefficient

#### Heterogeneity analysis: gender

Parental favouritism significantly increased the probability of depression in women by 38.1% (*β* = 0.381; 95% CI = 0.178, 0.585), with mother’s favouritism (*β* = 0.407; 95% CI = 0.189, 0.625) and father’s favouritism (*β* = 0.258; 95% CI = 0.017, 0.500) having a greater impact on women than on men (Table S2 in the **Online Supplementary Document**).

## DISCUSSION

Using data from CHARLS 2011, 2013, 2015, 2018, and 2020, we explored the effect of parental favouritism in childhood on depression in old age. We found that parental favouritism increased the probability of developing depression, with socialising, exercising, and sleep duration playing mediating roles in this relationship. Women were more affected by this phenomenon than men. This study is, therefore, the first in China to generate evidence on the link between childhood parental favouritism and depression in old age. We found that around 17% of the sample had experienced parental favouritism during childhood. Previous research identified favouritism in families of Turkey and the USA [20], suggesting it to be common across cultures. Previous studies have found that experiencing parental favouritism during childhood significantly increases the probability of developing depression in adulthood [21,22]. Using data from a five-wave follow-up period and a larger analytical sample, we observed that the negative effects of childhood parental favouritism persist into old age. This may be due to the failure of caregivers of favoured children to meet the basic emotional and psychological needs of non-favoured children, and unloved and uncared for children being likely to exhibit avoidant attachment traits in adulthood, and consequently more prone to depression [23,24]. In addition, neglected children have more negative expectations about emotions and relationships [24], which increases their risk of developing depression, with the condition persisting into old age. Our analyses showed father’s favouritism had a lower effect on developing depression than mother’s favouritism. This may be because mothers tend to take care of family members and have more interaction with children [25], resulting in this greater effect [21,26].

Here, we incorporated the mediating role of lifestyle and gender stratification to further explore the mechanism of action of childhood parental favouritism. Studies have shown that social contact and participation were important ways to alleviate mental health problems in the elderly [27,28], and that this could produce a continuous effect [29]. Simultaneously, we found a mediating role of exercising and sleep duration between parental favouritism and depression, possibly due to the fact that these activities helps promote mental health in older adults, which may be attributed to the fact that such healthy lifestyles help alleviate the negative impacts caused by adverse childhood experiences [30]

Furthermore, we observed differences in gender in terms of the effect of parental favouritism on depression after old age. This is consistent with previous research in Saudi Arabia, where men and women perceive parental favouritism differently [12]. Specifically, our findings suggest that parental favouritism have less effect on the probability of developing depression in men than in women. This may be explained by the fact that women tend to generate more emotional information than men and are more sensitive to their perception of others’ attitudes towards them [31,32].

Our study has several strengths. First, it is the first to explore the effect of parental favouritism on depression in old age in China, and thus provides empirical evidence for related interventions. Second, we identified a mediating factor in the effect of parental favouritism on depression, pointing to the importance of healthy lifestyle as a strategy for alleviating depression in old age. Third, we explored the differences between men and women in how parental favouritism impacts depression in old age, which could likewise help inform gender-specific interventions.

However, there are some shortcomings to this study. First, the measurement of childhood parental favouritism experiences depends on respondents’ memories, which may be subject to recall bias. Previous studies have faced the same problem and endogeneity, in that if an adult child suffers from depression, they may be more likely to perceive unequal treatment by parents [21]. However, we note that many previous studies have relied on self-reported retrospective methods to assess respondents’ adverse childhood experiences [33]. Second, we drew our data from five follow-up surveys over a 10-year period. Future research should explore this association using more robust study designs, larger sample sizes, and longer follow-up periods.

## CONCLUSIONS

Using data from five follow-up surveys conducted over a 10-year period, we observed that individuals who experienced parental favouritism in childhood were more likely to experience depression in old age. Promoting socialising, exercising, and sleep duration in old age could mitigate the negative effects of parental favouritism on depression. Lastly, we found that parental favouritism had a greater impact on women. These findings point to a need for mental health screening and intergenerational family counselling for older persons who experienced parental favouritism in childhood, with a focus on the promotion of healthy lifestyles alleviate the related psychological trauma.

## Additional material

Online Supplementary Document

## References

[1] Cavdar VC Ballica B Aric M Karaca ZB Altunoglu EG Akbas F Exploring depression, comorbidities and quality of life in geriatric patients: A study utilizing the geriatric depression scale and WHOQOL-OLD questionnaire. BMC Geriatr. 2024;24:687. 10.1186/s12877-024-05264-y 39143531 PMC11325729

[2] Bögels SM Brechman-Toussaint ML Family issues in child anxiety: Attachment, family functioning, parental rearing and beliefs. Clin Psychol Rev. 2006;26:834–56. 10.1016/j.cpr.2005.08.001 16473441

[3] McLeod BD Weisz JR Wood JJ Examining the association between parenting and childhood depression: A meta-analysis. Clin Psychol Rev. 2007;27:986–1003. 10.1016/j.cpr.2007.03.001 17449154

[4] Knappe S Beesdo-Baum K Wittchen HU Familial risk factors in social anxiety disorder: Calling for a family-oriented approach for targeted prevention and early intervention. Eur Child Adolesc Psychiatry. 2010;19:857–71. 10.1007/s00787-010-0138-0 20922550

[5] Brody LR Copeland AP Sutton LS Richardson DR Guyer M Mommy and Daddy like you best: Perceived family favouritism in relation to affect, adjustment and family process. J Fam Ther. 1887;20:269–91. 10.1111/1467-6427.00087

[6] Solmeyer AR McHale SM Parents’ differential treatment of adolescent siblings in African American families. Fam Process. 2017;56:171–88. 10.1111/famp.12166 26198081 PMC5513888

[7] Luo R Chen F Yuan C Ma X Zhang C Parent-child discrepancies in perceived parental favoritism: Associations with children’s internalizing and externalizing problems in Chinese families. J Youth Adolesc. 2020;49:60–73. 10.1007/s10964-019-01148-2 31889229

[8] Feinberg M Hetherington EM Differential parenting as a within-family variable. J Fam Psychol. 2001;15:22–37. 10.1037/0893-3200.15.1.22 11322082

[9] Suitor JJ Sechrist J Plikuhn M Pardo ST Gilligan M Pillemer K The role of perceived maternal favoritism in sibling relations in midlife. J Marriage Fam. 2009;71:1026–38. 10.1111/j.1741-3737.2009.00650.x 20104251 PMC2810864

[10] Bedford VH Memories of parental favoritism and the quality of parent-child ties in adulthood. J Gerontol. 1992;47:S149–55. 10.1093/geronj/47.4.S149 1624709

[11] Zervas LJ Sherman MF The relationship between perceived parental favoritism and self-esteem. J Genet Psychol. 1994;155:25–33. 10.1080/00221325.1994.9914755 8021620

[12] Moharib NI Effects of parental favoritism on depression and aggression in Saudi Arabian adolescents. Soc Behav Personal. 2013;41:1497–510. 10.2224/sbp.2013.41.9.1497

[13] Jiang W, Sun Z, Ma C. Effects of parental favoritism in childhood on depression among middle-aged and older adults: Evidence from China. In: Holl A, Chen J, Guan G, editors. Proceedings of the 2022 5th International Conference on Humanities Education and Social Sciences (ICHESS 2022); 14–16 October 2022; Chongqing, China. Dordrecht, Netherlands: Atlantis Press; 2022. p. 646–660.

[14] Wang D Zhao Y The Relationship between adverse family experiences during childhood and self-rated health outcome in adulthood. Soc Work Public Health. 2022;37:342–55. 10.1080/19371918.2021.2013384 34933661

[15] Ni Y Tein JY Zhang M Yang Y Wu G Changes in depression among older adults in China: A latent transition analysis. J Affect Disord. 2017;209:3–9. 10.1016/j.jad.2016.11.004 27866046

[16] Guo J Guan L Fang L Liu C Fu M He H Depression among Chinese older adults: A perspective from Hukou and health inequities. J Affect Disord. 2017;223:115–20. 10.1016/j.jad.2017.07.032 28753468

[17] Liu Y Diao L Xu L The impact of childhood experience of starvations on the health of older adults: Evidence from China. Int J Health Plann Manage. 2021;36:515–31. 10.1002/hpm.3099 33331669

[18] Lin L Cao B Chen W Li J Zhang Y Guo VY Association of adverse Childhood experiences and social isolation with later-life cognitive function among adults in China. JAMA Netw Open. 2022;5:e2241714. 10.1001/jamanetworkopen.2022.41714 36367722 PMC9652754

[19] Wang Q Association of childhood intrafamilial aggression and childhood peer bullying with adult depressive symptoms in China. JAMA Netw Open. 2020;3:e2012557. 10.1001/jamanetworkopen.2020.12557 32749469 PMC7403920

[20] Con G Suitor JJ Rurka M Gilligan M Adult children’s perceptions of maternal favoritism during caregiving: Comparisons between Turkey and the United States. Res Aging. 2019;41:139–63. 10.1177/0164027518785407 29991335

[21] Pillemer K Suitor JJ Pardo S Henderson C Jr Mothers’ differentiation and depressive symptoms among adult children. J Marriage Fam. 2010;72:333–45. 10.1111/j.1741-3737.2010.00703.x 20607119 PMC2894713

[22] Suitor JJ Gilligan M Peng S Jung JH Pillemer K Role of perceived maternal favoritism and disfavoritism in adult children’s psychological well-being. J Gerontol B Psychol Sci Soc Sci. 2017;72:1054–66. 26443015 10.1093/geronb/gbv089 PMC5927001

[23] Zhang Y Liao H Gu J Wang J Anxiety and depression related to childhood maltreatment in teenagers: Comparing multiple individual risk model, cumulative risk model and latent profile analysis. Child Abuse Negl. 2022;128:105630. 10.1016/j.chiabu.2022.105630 35413546

[24] Guler D Childhood psychological maltreatment and depressive symptoms: Parallel-serial mediating effects of certain psychological factors. Curr Psychol. 2022;41:4183–93. 10.1007/s12144-021-02182-9

[25] Xu L Liu L Li Y Liu L Huntsinger CS Parent-child relationships and Chinese children’s social adaptations: Gender difference in parent-child dyads. Pers Relatsh. 2018;25:462–79. 10.1111/pere.12254

[26] Jensen AC Whiteman SD Fingerman KL Birditt KS “Life still isn’t fair”: Parental differential treatment of young adult siblings. J Marriage Fam. 2013;75:438–52. 10.1111/jomf.12002 24833808 PMC4018724

[27] Okura M Ogita M Yamamoto M Nakai T Numata T Arai H More social participation is associated with less dementia and depression in Japanese older adults irrespective of physical frailty. Eur Geriatr Med. 2014;5:S114. 10.1016/S1878-7649(14)70275-X

[28] Tennant C Work-related stress and depressive disorders. J Psychosom Res. 2001;51:697–704. 10.1016/S0022-3999(01)00255-0 11728512

[29] Chiao C Weng LJ Botticello AL Social participation reduces depressive symptoms among older adults: an 18-year longitudinal analysis in Taiwan. BMC Public Health. 2011;11:292. 10.1186/1471-2458-11-292 21569285 PMC3103460

[30] Bloise SM Johnson MK Memory for emotional and neutral information: gender and individual differences in emotional sensitivity. Memory. 2007;15:192–204. 10.1080/09658210701204456 17534112

[31] Nurius PS Green S Logan-Greene P Borja S Life course pathways of adverse childhood experiences toward adult psychological well-being: A stress process analysis. Child Abuse Negl. 2015;45:143–53. 10.1016/j.chiabu.2015.03.008 25846195 PMC4470711

[32] Salokangas RKR From T Luutonen S Hietala J Adverse childhood experiences leads to perceived negative attitude of others and the effect of adverse childhood experiences on depression in adulthood is mediated via negative attitude of others. Eur Psychiatry. 2018;54:27–34. 10.1016/j.eurpsy.2018.06.011 30041073

[33] Kong J Homan KJ Goldberg J Longitudinal trajectories of adult sibling relationship quality and psychological well-being: The effect of childhood maltreatment. Fam Relat. 2024;73:891–904. 10.1111/fare.12945

