# Supplementary material for: Association of parental favouritism in childhood and depression in old age: a longitudinal survey
Source: J Glob Health. 2025 Dec 22;15:04343. doi: 10.7189/jogh.15.04343 (PMC12720492; doi:10.7189/jogh.15.04343)
Supplement: Online Supplementary Document [file jogh-15-04343-s001.pdf]

Supplement to: Li D, Li Z, Zhang W, Ge H, Su M, Guo X. Association of parental favouritism in childhood and depression in old age: a longitudinal survey. J Glob Health. 2025;15:04343.

Table S1. Outline of JoGH guideline items

JoGH guideline item	Author's Response
1. Please list all papers published by each co-author in previous 3 years that were based on secondary analysis of a big data repository	**Li, D., Guo, X., Zhang, W**., Li, W., Zhang, T., Liu, Z., **Su, M., & Li, Z**. (2025). The association between childhood hunger experiences and health in middle and old age: a longitudinal study over 10 years. BMC public health, 25(1), 193. https://doi.org/10.1186/s12889-025-21345-y<br><br>**Zhang, W., Su, M., Li, D., Guo, X., Li, Z.,** Zhang, T., & Hu, Z. (2024). The relationship between childhood hunger experiences and activities of daily living disability: a mediating role of depression. BMC public health, 24(1), 3050. https://doi.org/10.1186/s12889-024-20041-7<br><br>Yang, F., **Su, M.**, Zhang, T., **Zhang, W.**, Liu, Z., & Li, H. (2025). The impact of contracted family doctor services on social adaptability among middle-aged and older adults in rural China: the mediating role of regular health management. BMC public health, 25(1), 3342. https://doi.org/10.1186/s12889-025-24392-7<br><br>**Su, M.**, Zhang, T., **Zhang, W.**, **Li, Z.**, & Fan, X. (2024). Decomposition analysis on the equity of health examination utilization for the middle-aged and elderly people in

	China: based on longitudinal CHARLS data from 2011 to 2018. BMC public health, 24(1), 998. https://doi.org/10.1186/s12889-024-18068-x<br><br>**Li, D., Su, M., Guo, X.**, Liu, B., & Zhang, T. (2023). The association between chronic disease and depression in middle-aged and elderly people: The moderating effect of health insurance and health service quality. Frontiers in public health, 11, 935969. https://doi.org/10.3389/fpubh.2023.935969<br><br>**Zhang, W., Su, M., Li, D.**, Zhang, T., & Li, W. (2023). Catastrophic health expenditure and its inequality in rural China: based on longitudinal data from 2013 to 2018. BMC public health, 23(1), 1861. https://doi.org/10.1186/s12889-023-16692-7
2. Please explain the key elements of your study design and the use of the available datasets that make your study an original scientific contribution	This study aims to explore the association of parental favoritism in childhood and depression in old age, while attempting to identify the mechanism of action underlying this relationship and the gender differences in parental favoritism.<br>The China Health and Retirement Longitudinal Study (CHARLS) has conducted a 10-year follow-up survey on middle-aged and older adults. Specifically, through the Life Course Survey of Chinese Residents, it has documented the life experiences of CHARLS respondents since birth — a feature that facilitates our investigation into how the respondents' childhood experiences exert an influence on their health and life in old age.<br>This study yields the following findings: First, it identifies a significant correlation between parental favoritism in childhood and depression in old age. Second, parental favoritism affects late-life depression by acting on healthy lifestyles such as socialising, exercising, and sleep duration. Finally, the effect of parental favoritism on depression in old age exhibits gender heterogeneity.

3. Please list all publications that addressed similar research questions in the same dataset and indicate where you cited them in your paper	Jiang W, Sun Z, Ma C. Effects of parental favoritism in childhood on depression among middle-aged and older adults: Evidence from China. In: Holl A, Chen J, Guan G, editors. Proceedings of the 2022 5th International Conference on Humanities Education and Social Sciences (ICHESS 2022); 14–16 October 2022; Chongqing, China. Dordrecht, Netherlands: Atlantis Press; 2022. p. 646–660. (We cited this article in the Introduction. REFERENCES 13)
4. Please explain how you addressed multiple testing through an appropriately rigorous statistical threshold and indicate this in the methods section	In this study, the independent variable is parental favoritism, and the dependent variable is depression. The mediating variables include smoking (no = 0, yes = 1), drinking (no = 0, yes = 1), exercising (no = 0, yes = 1), socialising (no = 0, yes = 1), sleep duration (continuous variable), The control variables are gender (women = 0, men = 1), age (continuous variable), income (continuous variable), area of residence (rural = 0, urban = 1), marital status (unmarried = 0, married = 1), educational level completed (primary school or below = 0, middle school or above = 1), chronic disease (no = 0, yes = 1), and health insurance (no = 0, yes = 1).<br>Since the dependent variable was categorical, we used logistic regression analysis to investigate the relationship of parental favourism and depression, and utilised bootstrapping to analyse the mediating role of smoking, drinking, socialising, exercising, and sleep duration therein. For descriptive statistics, we expressed categorical variables as counts and percentages, and continuous variables as means and standard deviations (SDs). We performed the analyses in Stata, version 15.1 (Stata Corp, College Station, Texas, USA). The significance level for all hypothesis tests was set at 0.05.
5. Please declare to what extent have AI chatbots been used in developing your paper and to which parts of the paper did they contribute	No artificial intelligence chatbots were employed in the drafting of this thesis.

Table S2. Heterogeneity analysis of the effects of parental favouritism on depression

Variables	Women		Men	
	Depression		Depression	
	β (95% CI)	*P*-value	β (95% CI)	*P*-value
Parental favouritism	0.381 (0.178, 0.585)	0.000		
Parental favouritism			0.206 (−0.032, 0.443)	0.090
Mother's favouritism	0.407 (0.189, 0.625)	0.000		
Mother's favouritism			0.141 (−0.117, 0.400)	0.283
Father's favouritism	0.258 (0.017, 0.500)	0.036		
Father's favouritism			0.222 (−0.044, 0.488)	0.103

CI – confidence interval
